# Daxx and TCF4 interaction links to oral squamous cell carcinoma growth by promoting cell cycle progression via induction of cyclin D1 expression

**DOI:** 10.1007/s00784-015-1536-y

**Published:** 2015-07-24

**Authors:** Gu-Jiun Lin, Yen-Sung Huang, Chih-Kung Lin, Shing-Hwa Huang, Hsiu-Ming Shih, Huey-Kang Sytwu, Yuan-Wu Chen

**Affiliations:** Department of Biology and Anatomy, National Defense Medical Center, Taipei, Taiwan; Institute of Biomedical Sciences, Academia Sinica, Taipei, Taiwan; Department of Pathology, Taipei Tzu Chi general Hospital, New Taipei City, Taiwan; Department of General Surgery, Tri-Service General Hospital, National Defense Medical Center, Taipei, Taiwan; Department and Graduate Institute of Microbiology and Immunology, National Defense Medical Center, Taipei, Taiwan; Department of Oral and Maxillofacial Surgery, Tri-Service General Hospital, Taipei, Taiwan; School of Dentistry, National Defense Medical Center, 325 Cheng-Kung Road, Section 2, Nei-Hu, Taipei, 11490 Taiwan

**Keywords:** OSCC, Daxx, TCF4, Cyclin D1, Cell cycle

## Abstract

**Objectives:**

Death domain-associated protein (Daxx) has been recently implicated as a positive factor in ovarian cancer and prostate cancer, but the role of Daxx in oral squamous cell carcinoma (OSCC) has never been addressed. Herein, we investigate the expression and function of Daxx in OSCC.

**Materials and methods:**

RT-quantitative PCR, Western blotting, and immunohistochemistry were used to evaluation of the expression of Daxx in human OSCC cell lines and clinical surgical specimens. Short hairpin RNA targeting Daxx was transduced by lentivirus infection to knockdown the expression of Daxx in SAS and SCC25 cell lines, and the influence of this knockdown was evaluated by analyzing the growth and the cell cycle in transduced cells. Immunoprecipitation and sequential chromatin immunoprecipitation-quantitative PCR were used to analyze the associations between Daxx, TCF4, and cyclin D1 promoter. Xenograft tumor model was used to evaluate the in vivo tumorigenicity of Daxx in OSCC.

**Results:**

Daxx mRNA and protein expression are elevated in several OSCC cell lines and human OSCC samples in comparison to those in normal tissue. We further find that depletion of Daxx decreases OSCC cell growth activity through G1 cell cycle arrest. Daxx silencing reduces cyclin D1 expression via a Daxx-TCF4 interaction, whereas the Daxx depletion-mediated G1 arrest can be relieved by ectopic expression of cyclin D1. Moreover, we show that in OSCC clinical samples, the expression of Daxx is significantly correlated with that of cyclin D1.

**Conclusion:**

Our data demonstrate the importance of Daxx in regulation of cyclin D1 expression and provide the first evidence that Daxx exhibits tumor-promoting activity in OSCC.

**Clinical relevance:**

Daxx plays an important role in malignant transformation of OSCC and may serves as a target for cancer prevention and treatment.

## Introduction

Head and neck squamous cell carcinomas, including oral squamous cell carcinomas (OSCC), are the eighth most prevalent new cancer cases among men in the USA in 2014 [1]. The clinical outcome and prognosis for OSCC are dismal; more than 61 % of patients die of this disease or its complications within 5 years [2]. Thus, identifying cancer relative molecular markers for clinical histopathologic exams and finding the regulatory mechanisms of tumorigenicity in OSCC are necessary for development of potential therapeutics.

Daxx is a predominant nuclear protein that associates with several different subnuclear structures, including the PML nuclear body, heterochromatin, and nucleolus [3]. There are numerous reported functions for Daxx, acting as apoptosis regulator and transcription co-regulator in a SUMO binding-dependent or binding-independent manner [3–5]. Moreover, cooperation of Daxx with the chromatin remodeler ATRX is required for histone H3.3 deposition at the pericentromeric and telomere regions; H3.3 deposition facilitates transcript expression from pericentromeric regions [6]. Analysis derived from clinical samples in pancreatic neuroendocrine tumors (PanNETs) revealed a high ratio of inactivating-to-missense mutations in the coding region of Daxx [7]. Daxx has been recently implicated as a tumor promoting factor in ovarian cancer and prostate cancer, but the role of Daxx in OSCC has never been addressed [8, 9].

Although several Daxx-interacting proteins are involved in critical cellular pathways regarding P53 degradation, such as ubiquitin-specific-processing protease 7 (USP7) and mouse double minute 2 (Mdm2) [10, 11], it is still unclear whether Daxx has any role in tumorigenicity of OSCC. In the present study, we investigated the expression and the function of Daxx in OSCC. Our results provide the first evidence of elevated Daxx expression in both OSCC cell lines and clinical samples. We further demonstrated that Daxx silencing by RNAi reduces the cell growth of OSCC in vitro and in vivo. Daxx silencing reduces cyclin D1 expression and increases the fraction of cells in the G1 phase in OSCC, whereas ectopic expression of cyclin D1 eliminates the Daxx depletion-induced G1 cell cycle arrest. Together, our results suggest that Daxx silencing reduces OSCC cell growth through cyclin D1 downregulation.

## Materials and methods

### Tissue samples and ethics statement

All OSCC and matched normal tissues were obtained from 18 OSCC patients in the Department of Oral and Maxillofacial Surgery at Tri-Service General Hospital, Taiwan. The clinical and histological characteristics of the surgical specimens were presented in an additional Table 1. The experiments were under-taken following the approval by the Ethical Committee of Tri-Service General Hospital IRB protocol 096-05-002-I and 02-101-05-082, and written informed consents were obtained from all patients. The animal experiments were under-taken following the approval by the Institutional Animal Care and Use Committee of National Defense Medical Center IACUC protocol 11-084. All efforts were made to minimize the suffering of experimental animals.

**Table 1:** Clinical and histological characteristics of the 25 cases of primary human oral squamous cell carcinoma used in immunohistochemical analysis

Case No.	Gender	Age	Anatomic site	Stage	Histological grade
1	Male	63	Anterior pillar	I	Moderate
2	Male	37	Buccal	IVa	Poor
3	Male	44	Buccal	III	Poor
4	Male	66	Buccal	III	Moderate
5	Male	47	Buccal	IVa	Moderate
6	Male	44	Buccal	IVa	Moderate
7	Male	52	Buccal	IVa	Poor
8	Male	60	Gingiva	IVa	Moderate
9	Female	42	Gingiva	I	Moderate
10	Male	82	Gingiva	I	Moderate
11	Female	75	Gingiva	IVa	Well
12	Male	71	Lip	II	Moderate
13	Male	52	Mandible	IVb	Moderate
14	Male	57	Mouth floor	IVa	Poor
15	Male	39	Mucosa	IVa	Moderate
16	Male	48	Oral cavity	III	Poor
17	Male	45	Soft palate	III	Moderate
18	Male	40	Tongue	I	Well
19	Male	47	Tongue	IVa	Moderate
20	Male	44	Tongue	IVa	Poor
21	Male	52	Tongue	I	Moderate
22	Male	70	Tongue	I	Moderate
23	Female	74	Tongue	III	Moderate
24	Male	47	Tongue	II	Moderate
25	Female	48	Tongue	II	Poor

### Cells, siRNA, plasmids, and transfection

293T, WI38, and SCC25 cell lines were obtained from the American Type Culture Collection. The 293T and SCC25 cell lines used have been previously described in detail [5, 12]. Cell line WI38 was maintained in a minimal amount of essential medium supplemented with 10 % fetal bovine serum (FBS, non-essential amino acids, 1 mM sodium pyruvate, 2 mM L-glutamine, and antibiotics). The SAS cell line was provided by Dr. Jeng-Fan Lo and was grown in high glucose DMEM with 10 % FBS, as described [13]. The specific siRNA oligonucleotides used against Daxx and for negative controls were synthesized by Life Technologies [14]. The lentivirus plasmid expressing shLuc and shDaxx were obtained from the RNAi consortium at Academia Sinica. The shDaxx nucleotide sequences corresponded to Daxx coding sequence 1585-1605. Cyclin D1 plasmid was donated by Dr. William Hahn (Addgene plasmid no. 9050). Plasmids were isolated using a GenElute HP EndoFree Plasmid Maxiprep Kit (Sigma, St. Louis, MO, USA), and transfection was performed with a PolyJet (SignaGen Laboratories Ijamsville, MD, USA), according to the manufacturer’s instructions.

### Immunoprecipitation and Western blotting assays

Cells were lysed directly in an RIPA buffer containing 50 mM Tris (pH 7.8), 0.15 M NaCl, 5 mM EDTA, 0.5 % Triton X-100, 0.5 % NP-40, 0.1 % sodium deoxycholate, and protease inhibitor mixture (Sigma). The relative protein concentration in the supernatants was determined using a BCA protein assay kit (Thermo Scientific, Rockford, USA). For immunoprecipitation, 400 μg of protein lysates were incubated with specific antibodies. For each lane of 8 to 10 % SDS–PAGE gel, 40 μg protein of cell lysates were loaded, separated, and transferred onto polyvinyldifluoride (PVDF) membrane (Millipore, Bedford, MA, USA). The membranes were then probed using specific antibodies against Daxx (Sigma, D7810), cyclin D1 (Santa Cruz Biotech, Santa Cruz, CA, USA, sc-718), and β-actin (Sigma, A5441).

### RT-quantitative PCR

Total RNA was extracted from these cells using TRIzol (Life Technologies, Carlsbad, CA, USA) or Total RNA Miniprep Purification kit (GeneMark, Taipei, Taiwan). Five micrograms of RNA from each sample were then reverse transcribed using Superscript III Reverse Transcriptase (Life Technologies). RT-quantitative PCR (RT-qPCR) was performed using SYBR Green PCR master mix (Life Technologies) and an ABI 7500 sequence detection system (Life Technologies). The RT-qPCR primers used were as follows: *Daxx* forward, 5′-TGC AGA CAC CCC CGA AGC CT-3′; *Daxx* reverse primer, 5′-TGC CAT TCC ACT AGG GCC CTC A-3′; *GAPDH* forward, 5′-TCT TTT GCG TCG CCA GCC GAG-3′; *GAPDH* reverse primer, 5′-TGA CCA GGC GCC CAA TAC GAC-3′; *cyclin D1* forward primer, 5′-TGT GAC CCG GAC TGC CTC CG-3′; and *cyclin D1* reverse primer, 5′-GCG CAG GCT TGA CTC CAG CA-3′.

### Cell cycle analysis

Infected cells and transfected cells were harvested for the indicated periods, washed with ice-cold PBS, fixed overnight with 70 % ethanol at 4 °C, and then suspended in 500 μL PBS. After adding 10 μL RNase A (Sigma, 10 mg/mL), cells were allowed to stand at 37 °C for 30 min, stained with 10 μL 7-AAD (Sigma, 1 mg/mL), and then analyzed by flow cytometry. Flow cytometric analysis was performed using FACSCaliber (BD Biosciences, San Jose, CA, USA), and the data were analyzed using CellQuest software (BD Biosciences).

### Lentivirus production and infection

The replication deficient lentivirus was prepared by transfecting 293T cells with plasmids using a PolyJet (SignaGen Laboratories). Viral supernatants were harvested 72 h after transfection, clarified through centrifugation, filtered, and stored in aliquots at −80 °C. Cells were infected by lentivirus supernatants in the presence of 7.5 μg/mL Polybrene (Sigma) for 24 h.

### Xenograft tumor model

Eight-week-old NOD.CB17 *Prkdc*^*scid*^/J (National Laboratory Animal Center, Taiwan) mice were maintained in microisolator in pathogen free conditions. Five mice per group, which were randomly assigned, were injected subcutaneously with either shLuc or shDaxx lentivirus infected SAS cells. The sizes of the transplanted tumors were measured with gauged calipers every 3 days, and the tumor volumes were calculated using the following formula: *V* = 1/2 × (length × width^2^). At the end of treatment, the mice were euthanized, and the tumors were removed, weighed, and photographed.

### Immunohistochemistry

Parraffin sections were deparaffinized with xylene and rehydrated with a serial grade of alcohol. Epitope retrieval was carried out in a 10-mM citrate buffer (pH 6.0) water bathed at 90 °C on a hot plate for 18 min. After inactivation of endogenous peroxidase with H_2_O_2_, these slides were then incubated with specific antibodies against Daxx (Sigma, D7810) or cyclin D1 (Sigma, HPA027801) for 1 h at room temperature. Immunostaining was performed according to standard procedures. Envision plus kit (DAKO, Carpinteria, CA, USA) was used as a secondary reagent. Stainings were developed using DAB (brown precipitate). Slides were counterstained with hematoxylin and visualized by light microscopy. All immunostains were evaluated by two pathologists. Staining percentage was scored by counting the numbers of staining positive and calculating the percentage of positive cells. The scores of staining percentage was defined as 0 (0 %), 1 (1–25 %), 2 (26–50 %), 3 (51–75 %), and 4 (76–100 %). Staining intensity was determined by estimating the signal density and scored as 0 (no detectable stain), 1 (weak staining detected at intermediate to high power), 2 (moderate detected at low to intermediate power), to 3 (strong detected at low power). The final immunoreactivity score was scored by multiplying the staining percentage of positive cells by the staining intensity. Sample scores of 7–12 were defined as having high immunoreactivity, and sample scores of 0–6 were defined as having low immunoreactivity.

### Sequential chromatin immunoprecipitation-quantitative PCR assay

Chromatin immunoprecipitation-quantitative PCR (ChIP-qPCR) was performed as previously described [5]. ChIP-qPCR product was analyzed by quantitative real-time PCR using the Applied Biosystem 7500 Real-Time PCR System. A fraction (1 %) of the sonicated chromatin was set aside as input control before antibody affinity manipulations. Percent input was calculated by $100\kern0.5em \times \kern0.5em {2}^{\left({\mathrm{Ct}}^{\mathrm{adjusted}\ \mathrm{Input}}-{\mathrm{Ct}}^{\mathrm{IP}}\right)}$. Primers used to amplify DNA fragments containing the TCF4 consensus site were 5′-AGG CGC GGC GGC TCA GGG ATG-3′ and 5′-ACT CTG CTG CTC GCT GCT ACT-3′ for the human cyclin D1 promoter [15].

### Statistical analysis

All analyses performed with SigmaPlot software. The two-tailed Students’s *t* test was used to evaluate the significance of the differences between two groups of data in all experiments. Values of *P* < 0.05 were considered significant.

## Results

### Daxx expression is frequently upregulated in both OSCC human samples and cell lines

**Fig. 1:**
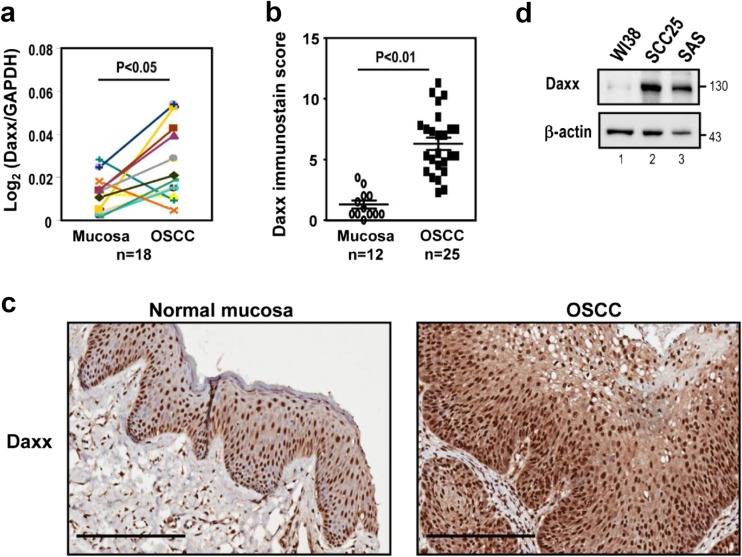
Daxx expression is frequently upregulated in OSCC clinical samples and OSCC cell lines. **a** Quantitative RT-PCR results from 18 pairs of matched normal mucosa tissue and OSCC tissues. **b** Immunohistochemitry of Daxx expression in a normal mucosa tissue and OSCC tissue. Statistical significance was ascertained by conducting Student’s *t* tests. **c** Positive nuclear immunolabeling of Daxx in normal mucosa tissue and OSCC tissue. *Scale bar* = 200 um. **d** Western blots of Daxx expression in a normal cell line (WI38) and two human OSCC cell lines (SCC25 and SAS). β-actin was used as a loading control

To examine whether Daxx expression were dysregulated in human OSCC samples, we measured the *Daxx* mRNA levels in 18 pairs of OSCC tissues and their matched normal mucosa tissues. Among the 18 patients, 14 (78 %) patients showed higher *Daxx* RNA level in the OSCC tissues than that in adjacent normal mucosa tissues (Fig. 1a). We further analyzed Daxx protein in a set of human OSCC samples and observed that Daxx expression was significantly higher in OSCC tissues than in normal mucosa tissues (Fig. 1b). Notably, Daxx was located in nuclei of normal mucosa tissue and OSCC tissue (Fig. 1c). The results indicated that higher staining intensity and percentage of Daxx positive cells in tumor tissue compared to normal tissue (Fig. 1c). We also investigated the expression levels of Daxx in OSCC cell lines (SCC25 and SAS) and a normal cell line (WI38). SCC25 and SAS cell lines expressed higher levels of Daxx than WI38 cell line (Fig. 1d). Taken together, Daxx expression is frequently upregulated in both OSCC clinical samples and OSCC cell lines.

### Downregulation of Daxx reduces the growth of OSCC cells in vitro and in vivo

**Fig. 2:**
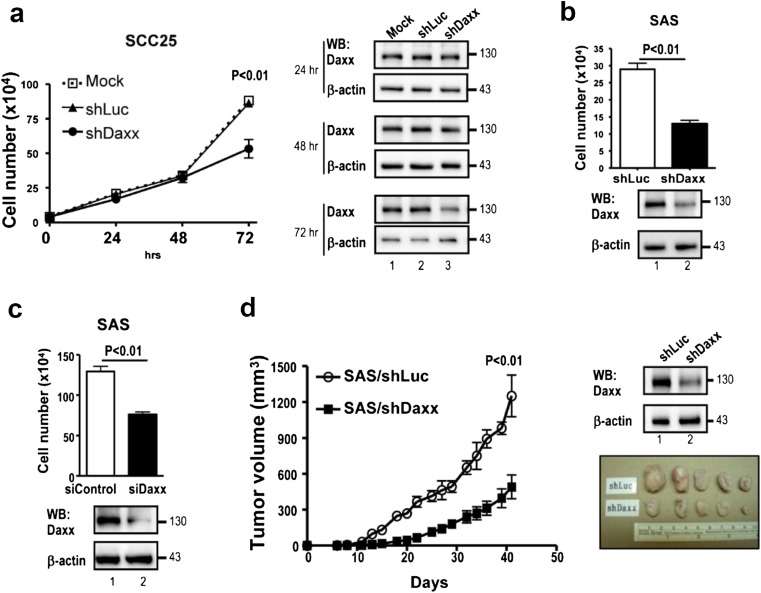
RNAi-mediated down-regulation of Daxx reduces the growth activity of OSCC cells in vitro and in vivo. **a** SCC25 and **b** SAS cells infected with shLuc and shDaxx lentivirus were counted at the indicated times. Infected cells were then subjected to Western blots with indicated antibodies. **c** SAS cells transfected with siControl and siDaxx were counted at day 3 after transfection and subjected to Western blots with indicated antibodies. **d** SAS cells infected with shLuc and shDaxx were subcutaneously xenografted into NOD/SCID mice (*n* = 5 mice per group). Data are represented as mean ± SEM. The tumor size was analyzed using Student’s *t* test at day 42 after tumor cell injection (*left panel*). *Top right panel*: Western blot of an aliquot of the infected cells before injection. *Bottom right panel*: tumors from SAS/shLuc or SAS/shDaxx were excised at day 42

The elevated Daxx expression in both OSCC cell lines and OSCC clinical samples led us to investigate whether endogenous Daxx affects the growth capability of OSCC cell. Lentivirus-mediated shRNA was used to knockdown the Daxx protein expression in both SCC25 and SAS OSCC cell lines. This shDaxx plasmid has been widely used to target Daxx mRNA in several studies [5, 16]. The results indicated that effective silencing of Daxx expression in SCC25 and SAS cells significantly reduces the cell number compared to control cells at day 3 after infection (Fig. 2a, b). Another siRNA oligo against Daxx in a different coding region was also used. Consistent with the lentivirus results, Daxx siRNA (siDaxx) decreases SAS cell viability compared to control cells at day 3 after transfection (Fig. 2c). Xenograft tumor experiments were used to extend the study of endogenous Daxx in tumorigenicity. The results indicated that shDaxx-infected cells significantly reduce tumor growth rates in NOD/SCID mice compared with shLuc-infected cells (Fig. 2d). Collectively, these results further support the notion that suppression of Daxx expression significantly decreases the tumor growth of OSCC.

### Daxx silencing reduces cyclin D1 expression via a Daxx-TCF4 interaction

**Fig. 3:**
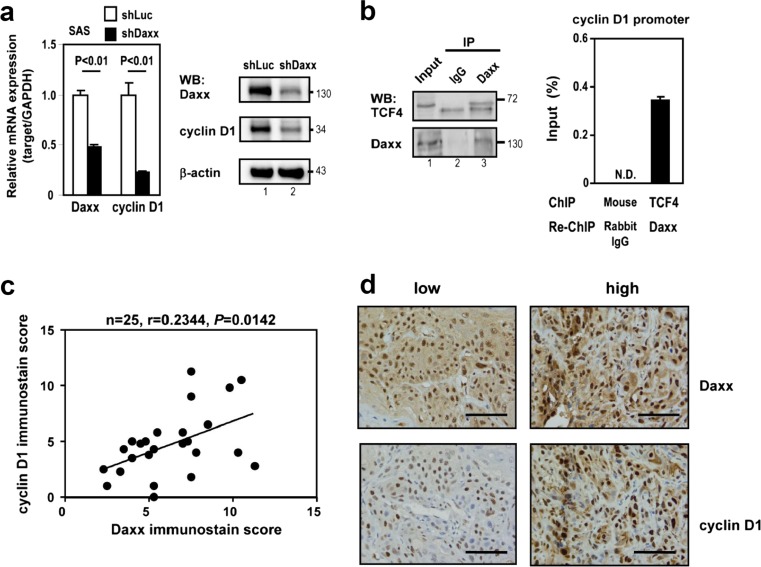
Daxx silencing reduces cyclin D1 expression via a Daxx-TCF4 interaction. **a** RT-qPCR and Western blot of cyclin D1 expression in Daxx shRNA-infected SAS cells. Data are presented as the mean ± SD from three experiments. Statistical significance was ascertained using Student’s *t* test. **b** Western blot and sequential chromatin immunoprecipitation (ChIP) assays were performed with antibodies against Daxx, TCF4, or IgG control. Input represents 5 % of the lysates used for Western blot and 1 % of the chromatin used for ChIP, respectively. Data are presented as the mean ± SD from three independent experiments. *N.D.* not detected. **c** A positive correlation between Daxx (*x* axis) and cyclin D1 (*y* axis) in OSCC samples using immunohistochemistry. *Each dot* corresponds to one sample. Statistical significance was ascertained using Regression analysis. **d** Representative photographs of Daxx- and cyclin D1-stained tumors collected from OSCC clinical samples with low and high staining

It has been previously reported that Daxx regulates the expression of cyclin D1 at the transcription level in colorectal cancer [17] and that cyclin D1 is important for OSCC growth activity [18]; we therefore tested whether Daxx can regulate cyclin D1 expression in OSCC using OSCC cell line. We found that suppression of Daxx by RNAi decreases the mRNA transcription and protein expression of cyclin D1 in SAS cells, an OSCC cell line (Fig. 3a). Previous studies indicated that expression of cyclin D1 is strongly dependent on β-catenin/TCF4-mediated transcriptional regulation [19]. We showed an endogenous interaction between Daxx and TCF4 in SAS cells using immunoprecipitation (IP) followed by Western blotting (Fig. 3b, left panel). Moreover, the association of Daxx and TCF4 was found on endogenous cyclin D1 promoter in SAS cells as evidenced by the presence of the TCF4-containing DNA fragment precipitated by anti-TCF4 antibody followed by sequential chromatin immunoprecipitation (ChIP) with anti-Daxx antibody but not by a control antibody (Fig. 3b, right panel). As we hypothesized that transcription of *cyclin D1* is regulated by Daxx, we therefore examined whether cyclin D1 is coexpressed with Daxx in human OSCC samples. We evaluated *Daxx* and *cyclin D1* expression in OSCC clinical samples by RT-qPCR and found a strong correlation between *Daxx* and *cyclin D1* expression (*r* = 0.7074, *P* = 0.0149, data not shown). Similar results were also observed from immunohistochemistry (Fig. 3c, *n* = 25). Accordingly, Daxx expression level was correlated with cyclin D1 level in different OSCC samples (Fig. 3d). Together, our results suggest that Daxx functions as a positive regulator in modulating *cyclin D1* expression via interacting with TCF4 in OSCC cells.

### Daxx silencing induces cyclin D1-mediated G1 arrest in OSCC cells

**Fig. 4:**
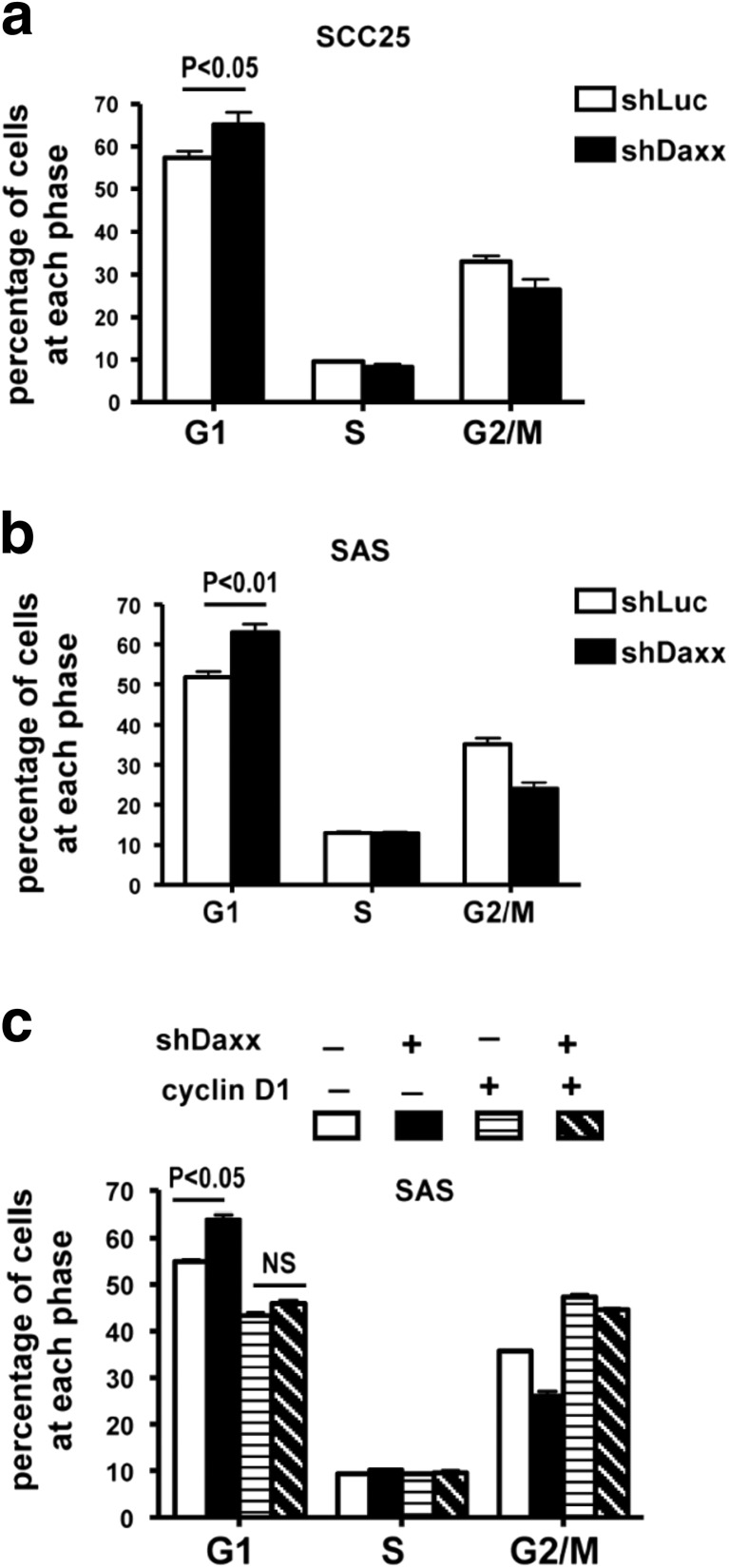
Daxx silencing induces cyclin D1-mediated G1 arrest in OSCC cells. **a** SCC25 cells and **b** SAS cells infected with lentivirus containing shLuc or shDaxx for 3 days were fixed and stained with 7-AAD for cell cycle analysis. **c** Flow cytometric cell cycle analysis of SAS cells transfected with plasmids as indicated after 3 day. Data are presented as the mean ± SD from three independent experiments. Statistical significance was ascertained by conducting Student’s *t* tests

Given that cyclin D1 plays a critical role in the G1 to S phase cell cycle transition [19], we expect that Daxx silencing should induce G1 arrest. We therefore examined the effects of Daxx silencing on cell-cycle progression. Indeed, suppressing Daxx in SCC25 and SAS cells enhanced the portion of cells in the G1 phase (Figs. 4a, b). To confirm this notion, cyclin D1 was ectopically expressed in Daxx-depleted SAS cells. Our results showed that ectopic expression of cyclin D1 eliminates Daxx depletion-induced G1 arrest (Fig. 4c). Taken together, Daxx regulates the growth activity of OSCC cells which may be associated with cyclin D1.

## Discussion

Aberration of cell cycle regulation accounts for many abnormal biological behavior of cancer cells. In this study, we show that Daxx silencing in OSCC cells increases the number of G1-arrested cells through downregulating cyclin D1 expression. Cyclin D1 is a vital protein that controls the transition from G1 to S phase and governs the rate of cell proliferation. Increase or upregulation of cyclin D1 has been reported in about 36 to 56 % of OSCC patients, and therefore, it acts as an important genetic event in OSCC [18]. Recently, Zhao et al. [20] indicated that cyclin D1 expression correlates with detrimental clinicopathological outcome and poor prognosis in OSCC. Thus, it is possible that Daxx may be used as a prognostic factor for OSCC. In the current study, we provide evidence for a possible link between Daxx expression level and the pathogenesis of OSCC. How the correlation between Daxx and cyclin D1 is involved in clinicopathological features and poor prognosis of OSCC needs to be dissected in the future.

In this current study, we have shown that Daxx silencing reduces tumor growth by suppressing cyclin D1 expression in OSCC. In line with this notion, a few recent studies indicate that Daxx may play a tumor promoting role in several cancer types. For example, Daxx destabilizes P53 via inhibition of Mdm2 ubiquitination in osteosarcoma and colorectal cancer cells [10]. In addition, Daxx potentiates TCF4/β-catenin-mediated transcriptional activation in colorectal cancer cells [17]. Furthermore, Daxx is frequently overexpressed in prostate cancer cells and the expression level is significantly correlated with prostate cancer stage [8, 21]. Moreover, Daxx silencing suppresses the growth activity of human ovarian cancer cells and mouse ovarian surface epithelial cells [9, 22]. Development of OSCC is a multi-step progressing process of transformation from healthy mucosa to invasive carcinoma. Each step is accompanied by specific genetic alterations, including inactivation of P53 and overexpression of cyclin D1 [23]. Since Daxx is a negative regulator of P53 and a positive regulator of cyclin D1, these facts support the notion that Daxx exerts a tumor promoting effect in OSCC or other types of cancer. However, these results are derived from cell models. It is important to explore the oncogenic contribution of Daxx in different cancer models using conditional gene knockout mice.

This is the first study to demonstrate the underlying mechanism in OSCC of how the expression of cyclin D1 is regulated by Daxx through an interaction with TCF4 on the cyclin D1 promoter. Even though the molecular basis of how Daxx enhances cyclin D1 expression is currently unclear, we speculate two possible scenarios. First, Daxx may recruit CBP to potentiate TCF4-mediated transactivation since Daxx could function as a coactivator through recruiting CBP in a cell-context-dependent manner [24]. Secondly, Daxx acts as a chaperone for the histone variant H3.3 which is enriched at centromeric heterochromatin, telomeres, and active genes. Therefore, Daxx may regulate transcription through loading H3.3 at regulatory regions [25]. These two possibilities are not mutually exclusive. Further studies are required to clarify the molecular mechanism of Daxx in enhancement of cyclin D1 expression.

In sum, we have discovered for the first time that the expression of Daxx mRNA and protein was upregulated in OSCC clinical samples as well as in OSCC cell lines. Moreover, knocking down Daxx inhibited cell growth through inducing G1 cell cycle arrest and decreased cyclin D1 expression. Hence, Daxx silencing may reduce tumorigenicity in OSCC. Thus, it is reasonable to speculate that Daxx plays an important role in malignant transformation of OSCC and serves as a potential target for cancer prevention and treatment. Further mechanistic studies may advance our knowledge of the role of Daxx in OSCC and cancer development.
